# Expression and mechanisms of interferon-stimulated genes in viral infection of the central nervous system (CNS) and neurological diseases

**DOI:** 10.3389/fimmu.2022.1008072

**Published:** 2022-10-17

**Authors:** Rui Lang, Huiting Li, Xiaoqin Luo, Cencen Liu, Yiwen Zhang, ShunYu Guo, Jingyi Xu, Changshun Bao, Wei Dong, Yang Yu

**Affiliations:** ^1^ Key Laboratory of Medical Electrophysiology, Ministry of Education & Medical Electrophysiological Key Laboratory of Sichuan Province, (Collaborative Innovation Center for Prevention of Cardiovascular Diseases), Institute of Cardiovascular Research, Southwest Medical University, Luzhou, China; ^2^ Department of Neurosurgery, The Affiliated Hospital of Southwest Medical University, Luzhou, China; ^3^ Department of Human Anatomy and Histoembryology, School of Basic Medical Sciences, Southwest Medical University, Luzhou, China; ^4^ Department of Pathology, People’s Hospital of Zhongjiang County, DeYang, China; ^5^ Sichuan Clinical Research Center for Neurosurgery, The Affiliated Hospital of Southwest Medical University, Luzhou, China; ^6^ Academician (Expert) Workstation of Sichuan Province, The Affiliated Hospital of Southwest Medical University, Luzhou, China; ^7^ Neurological diseases and brain function laboratory, The Affiliated Hospital of Southwest Medical University, Luzhou, China

**Keywords:** interferons, interferon-stimulated genes, central nervous system, interferon-stimulated gene 15, interferon-inducible tetrapeptide repeat protein 2, interferon-inducible transmembrane protein 3, viral infection, neurological diseases

## Abstract

Interferons (IFNs) bind to cell surface receptors and activate the expression of interferon-stimulated genes (ISGs) through intracellular signaling cascades. ISGs and their expression products have various biological functions, such as antiviral and immunomodulatory effects, and are essential effector molecules for IFN function. ISGs limit the invasion and replication of the virus in a cell-specific and region-specific manner in the central nervous system (CNS). In addition to participating in natural immunity against viral infections, studies have shown that ISGs are essential in the pathogenesis of CNS disorders such as neuroinflammation and neurodegenerative diseases. The aim of this review is to present a macroscopic overview of the characteristics of ISGs that restrict viral neural invasion and the expression of the ISGs underlying viral infection of CNS cells. Furthermore, we elucidate the characteristics of ISGs expression in neurological inflammation, neuropsychiatric disorders such as depression as well as neurodegenerative disorders, including Alzheimer’s disease (AD), Parkinson’s disease (PD), and amyotrophic lateral sclerosis (ALS). Finally, we summarize several ISGs (ISG15, IFIT2, IFITM3) that have been studied more in recent years for their antiviral infection in the CNS and their research progress in neurological diseases.

## Introduction

Interferons (IFNs) are a class of antiviral cytokines that are stimulated in response to the challenge of host defenses and are crucial for mobilizing the immune response to pathogens in vertebrates. Most virus-infected cells can produce IFNs, which are secreted to bind to their receptors on autologous or other cells and initiate a signaling cascade that leads to the induction of hundreds of interferon-stimulated genes (ISGs) to promote antiviral effects (1, 2). IFNs are divided into three classes (type I, type II, and type III) based on their sequence and cellular receptors. The type I IFN family comprises members encoded by multiple genes, including 13 highly homologous subtypes of IFN-α, and the other isoforms, such as IFN-β, IFN-ϵ, IFN-κ, IFN-ω, IFN-τ, IFN-δ, and IFN-ζ (3–6). The type II IFN class only contains IFN-γ, while the type III IFN family consists of four IFN-λ molecules, including IFN-λ1(IL-29), IFN-λ2 (IL-28A), and IFN-λ3 (IL-28B), as well as IFN-λ4 (7). Type I and III IFNs are considered the classical antiviral IFNs, while type II IFN has multiple roles in the innate and adaptive immune systems (1, 2, 7–10). Additionally, type III IFNs impact antiviral activity at anatomical barriers, such as the blood-brain barrier (BBB) and epithelial cell surfaces (11). Although type I and type III IFNs induce a similar subset of ISGs, differences in cell-type specificity and signaling kinetics result in distinct responses. In general, type I IFNs activate a more robust and rapid ISG response, whereas type III IFNs induce a slower response with lower levels of ISG expression (reviewed in 2). Traditionally, IFN signaling is involved in the induction of host defense-associated ISGs through the Janus tyrosine kinase (JAK)/signal transducer and activator of transcription (STAT) signal pathway (12–14). Type I and III IFNs activate JAK1 and tyrosine kinase 2 (TYK2), resulting in cytoplasmic STAT1 and STAT2 phosphorylation (15). After phosphorylation, STAT1 and STAT2 dimerize and translocate from the cytoplasm to the nucleus, forming the IFN-stimulated gene factor 3 (ISGF3) complex with interferon regulatory factor 9 (IRF9). ISGF3 further binds to interferon stimulatory response elements (ISREs) and stimulates the transcription of ISGs (16). Type II IFN activates JAK1 and JAK2, resulting in the formation of phosphorylated STAT1 (pSTAT1) homodimers known as γ-activated factors (GAF), which translocate to the nucleus and bind to γ-activated sequences (GAS) to induce transcription of ISGs (17). Some interferon regulatory factors (IRFs) such as IRF3 can induce ISGs directly in the absence of IFN production or collaboratively with other transcription factors such as IFN regulatory factor 7 (IRF7), IRF1, and nuclear factor kappa B (NF-κB) to induce type I IFN production (17–26). In addition, some ISGs encode factors that are involved in the IFN production or response pathway through positive or negative feedback loops (27, 28). For instance, the core retinoic acid-inducible gene I (RIG-I) and melanoma differentiation-associated gene 5 (MDA5), members of the mammalian RIG-I-like receptors (RLRs), are found in the cytosol of most cell types and are powerfully activated by IFNs in a positive feedback loop of RNA virus infection (27, 29). Interferon-responsive activation of interferon-induced protein with tetrapeptide repeats 1 (IFIT1, also known as ISG56) positively regulates the expression of RIG-I, MDA5, and IFIT2 (also known as ISG54) (30). Furthermore, IFN-stimulated gene 15 (ISG15) was found to negatively regulate the IFN signaling pathway by coupling to RIG-I (31, 32).

The IFN pathway provides essential protection to the central nervous system (CNS) against viral infections. It is instrumental in immune-related diseases such as allergic reactions, chronic inflammatory diseases, autoimmune diseases, transplant rejection, viral infections, and many more (33). For example, mice with a deficiency in the IFN-I receptor subunit 1 (*Ifnar1^-/-^*) are highly susceptible to various viral infections in multiple organs, including the CNS (34–36). *Ifnar1^-/-^* mice showed increased viral load after infection with Sindbis virus (SINV), and increased viral load in the CNS was associated with high susceptibility compared with wild-type (WT) mice (37). Several fatal cases of Herpes simplex encephalitis (HSE) in newborns were associated with defects in genes encoding signal transduction factors of the IFN pathway, such as Toll/interleukin-1 receptor domain-containing adaptor-inducing interferon-β (TRIF), TANK-binding kinase 1 (TBK-1), Toll-like receptor 3 (TLR3) or tumor necrosis factor receptor-associated factor 3 (TRAF3). These findings demonstrated the importance of the human IFN response to neurotropic viral infections (38–41). In addition to participating in natural immunity against viral infections, IFNs have been shown to constitute key factors in the neuroinflammatory network and make an essential contribution to the pathogenesis of neurodegenerative diseases such as Parkinson’s disease (PD), Alzheimer’s disease (AD), and amyotrophic lateral sclerosis (ALS) (42–45). The IFN signaling pathway was recently reported to be severely upregulated in AD patients and significantly correlated with disease severity (46–48). Activation of the IFN signaling pathway can induce the expression of hundreds of ISGs. Although ISGs are major antiviral effectors of the IFN response, the antiviral mechanisms of most ISGs have not been described until recently. The aim of this review is to provide a macroscopic overview of the characteristics of ISGs that restrict viral neural invasion and cellular expression of ISGs after viral infection of the CNS, as well as the expression characteristics of ISGs in neurological diseases (**Table 1**). In addition, substantial progress has been made in our understanding of individual ISGs (ISG15, IFIT2, IFITM3) in CNS viral infection and diseases in recent years (**Table 1**), providing an essential target for the development of novel antivirals and anti-neurological disease drugs.

**Table 1:** ISGs expression in antiviral infections, neuronal localization and neurological diseases.

ISGs	Produced by viruses and related stimuli	Specifically altered in CNS regions or cells	Neurological disorders and related models
ISG15	TMEV (49); HIV (50, 51); MHV-induced encephalitis (52);	Astrocytes and endothelial cells (49); BMECs (50); Microglia (51);	Post-traumatic brain injury ALS (53); ALS, model of cerebral ischemia, model of brain injury, model of chronic neuronal damage induced by the viral protein HIV gp120 (54); Ataxia capillaries (A-T) (55); Maternal immune activation (MIA) (56); AGS (57);
MX2	HIV (50, 51); Poly I:C (58);	BMECs (50); Microglia (51); Hippocampal dentate gyrus (58);	AGS (57);
Viperin	HIV (51); HSV-1 (59, 60); LGTV (61); TBEV (61);	Microglia (51); Neurons and astrocytes (59, 60);	AGS (57);
CH25H	HSV-1 (59, 60);	Neurons and astrocytes HSE (59, 60);	Multiple sclerosis (MS) (62);
IFITM3	SARS-CoV-2 (63); HCMV (64);	frontal cortex and choroid plexus (65);	The aging mouse (66); 5xFAD Alzheimer’s disease mouse model (66);
OAS2	HSV-1 (59, 60);	Neurons and astrocytes (59, 60);	
latent RNase (RNase L)	HSV-1 (59, 60);	Neurons and astrocytes (59, 60);	
PKR	HSV-1 (59, 60);	Neurons and astrocytes (59, 60);	
IFIT1	HIV (50, 51); VSV (67); EMCV (67); HSV-1 (59, 60); JEV (68); Poly I:C (30); HCMV (69); MHV (52);	BMECs (50); Neurons and astrocytes (59, 60); Microglia (68); Astrocytes (30, 69);	
GBP5	HIV (51);	Microglia (51);	
IFIT2	JEV (68); VSV (67, 70); WNV (71); EMCV (67); Sendai virus (SeV) (72); MHV-RSA59 (73); RABV (74, 75); Poly I:C (30, 58);	Microglia (68);	
Ifi27l2a	WNV (76);		
IFP35			MS (77);
IFI27			AGS (57);
IFIT3	RABV (74);		

## ISGs play an essential role in restricting viral neuroinvasion

**Figure 1:**
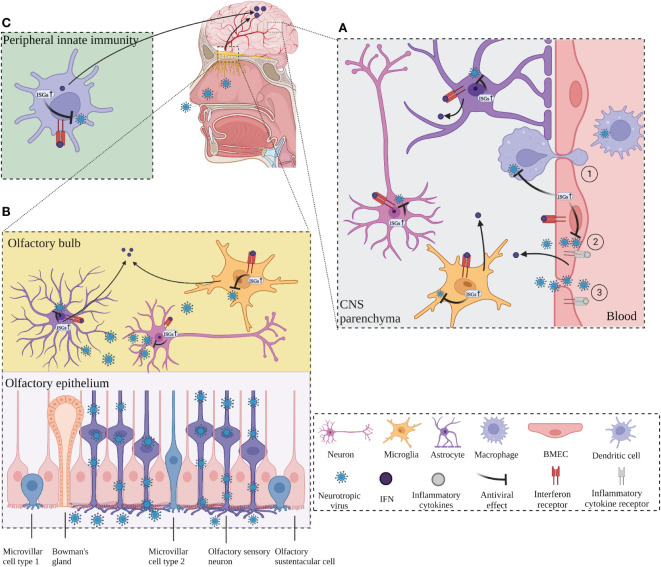
ISGs restrict viral neuroinvasion. **(A)** ISGs in BMECs, which constitute the blood-brain barrier (BBB), are activated by virus and inflammation, leading to an IFN response and activation of transcription of antiviral ISGs, followed by activation of microglia and astrocytes to release ISGs. BMECs deliver the released ISGs to macrophages to enhance the antiviral response and restrict viral invasion. ① Neurotropic virus crosses the BBB through “Trojan horse” strategy. ② Virus infects endothelial cells across the BBB. ③ Virus enters the CNS by destroying the BBB. **(B)** The virus invades the CNS retrogradely through the olfactory pathway. The virus invades the olfactory sensory neurons through the olfactory epithelium and retrogrades upward into the olfactory bulb (OB). Virus at OB activates neurons, microglia, and macrophages, which produces IFN response and induces transcription of ISGs, leading to antiviral response. Long-distance signaling of IFN at the OB activates the transcription of ISGs in the brain. **(C)** IFNs produced by peripheral antiviral response enter the CNS and activate the transcription of ISGs in advance and induces transcription of ISGs, leading to antiviral response. Long-distance signaling of IFN at the OB activates the transcription of ISGs in the brain. **(C)** IFNs produced by peripheral antiviral response enter the CNS and activate the transcription of ISGs in advance. Created with BioRender.com.

The CNS requires a complex and coordinated immune response to prevent neurological disorders, avoid excessive immune activation and inappropriate inflammatory response, and protect against invading pathogens such as viruses. Although the CNS is immune privileged and protected from toxic substances and pathogens carried in the blood by the BBB and the blood-cerebrospinal fluid barrier (BCSFB) (78), neurotropic viruses are capable of infecting the CNS and staying there for a long time, including herpes simplex virus type 1 (HSV-1), varicella zoster virus (VZV), Japanese encephalitis virus (JEV), West Nile virus (WNV), measles, rabies virus (RABV), poliovirus, and so on (79–81). Neurotropic viruses can spread through multiple pathways to penetrate the CNS. Some viruses can enter the CNS by infecting host immune cells in the periphery and using these cells as “Trojan horses” to carry them across the BBB, or the virus can directly infect endothelial cells, disrupt the BBB, or infect peripheral neuron axons and retrograde upward through the mechanism of axonal transport of cellular cargo, such as through the olfactory pathway (33, 79, 80) (**Figure 1**).

### ISGs prevent viruses from crossing the BBB

The structural integrity of the BBB is critical to enable the normal neurological function of the CNS and protection from damage from inflammation, virus, and other diseases. Brain microvascular endothelial cells (BMECs) are the structural and functional basis of the BBB and play a critical role in maintaining its normal integrity. One study reported that infection of BMECs by JEV did not affect cell viability but resulted in increased permeability of the endothelial monolayer due to inflammation caused by JEV infection that inhibited the expression of tight junction (TJ) proteins in BMECs, leading to enhanced BBB permeability (81). *In vitro* experiments with cultured mouse BMECs showed that IFN-λ signaling increased transendothelial resistance, reduced viral movement across the barrier, and modulated TJ protein localization (82). In addition, IFN-λ limits the opening of the BBB by reducing the production of inflammatory cytokines in primary astrocytes and microglia and inducing activation of the JAK/STAT pathway, leading to the production of ISGs (83). Inflammation can play an indirect antiviral role while disrupting the BBB, and in an *in vitro* model of the BBB with co-culture of astrocytes and BMECs, lipopolysaccharide (LPS) promoted the expression of type I IFN signaling-related proteins such as STAT1, STAT2, ISG15, and SAMHD1 in astrocytes, TNF-α and LPS also induce the production of ISGs (EIF2AK2, ADAR, TRIM25 and ISG15) in astrocyte cultures *in vitro* (84). BMECs have a critical function in the innate immunity of the BBB to human immunodeficiency virus (HIV) infection by causing activation of TLR3 in BMECs to induce phosphorylation of IRF3 and IRF7 (key regulators of the IFN signaling pathway) and trigger the production of endogenous IFN-β and IFN-λ, thereby significantly inhibiting viral replication (85). In addition, TLR3-activated human BMECs secrete exosomes that inhibit HIV replication by transferring antiviral factors, including several critical IFN-stimulated genes (ISG15, IFIT1, MX2) to macrophages (50). This suggests that human BMECs may help restore the antiviral status of HIV-infected macrophages, which may be a defense mechanism against neural invasion by HIV “Trojan horses” (**Figure 1A**).

### ISGs limit viral infection in the olfactory pathway

Numerous neurotropic viruses enter the CNS through infection of peripheral nerves, including olfactory neurons and sensory or motor neurons (86). WNV can enter the CNS by infecting sensory nerve endings or olfactory neurons or through the bloodstream (87). RABV and poliovirus transmit from muscle to somatic motor neurons in the spinal cord *via* the neuromuscular junctions (NMJs) (88). Olfactory pathways play an important role in the invasion of viruses into the CNS *via* peripheral nerves (**Figure 1B**). Detje et al. found that blocking the type I IFN pathway promoted the spread of vesicular stomatitis virus (VSV) from the olfactory bulb (OB) to the entire CNS, while local IFN response in the OB effectively controlled viral invasion of the CNS (89). IFN-induced viperin limits the replication of Langat virus (LGTV) in the OB in a region-specific manner (61). Long-range signaling of IFN-β released from infected neurons at the OB after VSV and cytomegalovirus (CMV) infection of the nasal mucosa upregulated the expression of ISGs in uninfected brain regions (90). Similar results were obtained from another investigation, which shows that after intranasal VSV infection, IFN expressed at the OB enters the brain to activate IFIT2 transcription in advance to act as an antiviral agent (67). The accumulation of microglia around the OB and their expression of ISGs form a natural immune barrier that is instrumental in limiting the spread of VSV in the CNS and preventing fatal encephalitis (91) (**Figure 1B**).

### ISGs are activated before viruses invade the CNS

Viral infections rapidly induce IFN in the periphery, which serves to protect most tissues from viral pathogenicity (92). Peripherally induced IFN response can induce ISGs in the brain. Peripheral IFN-α that crosses the BBB directly activates IFN-α/β receptor (IFNAR) signaling in microglia, leading to the upregulation of multiple ISGs (93, 94). As mentioned above, long-range signaling of IFN released from infected neurons at the OB after viral infection of the nasal mucosa upregulated the expression of ISGs (IFIT2, IFIT3, OAS, and MX1) in uninfected brain regions (67, 90). Lukasz et al. reported that adolescent mice injected with the viral mimic poly I:C significantly increased the expression of ISGs (IFIT2, PRKR, MX2, and IRF7) in the hippocampal dentate gyrus (58). Collectively, these findings suggest that early activation of ISGs in the CNS plays a crucial role in limiting viral infection of the CNS (**Figure 1C**).

## Antiviral response in the CNS: Cells, ISGs, and mechanisms

The vast majority of cell types in the CNS, comprising neurons, astrocytes, oligodendrocytes, CNS-associated macrophages (CAMs), ventricular epithelial cells, and vascular endothelial cells, are responsive to IFN (95). Activation of astrocytes and microglia in the brain has an essential role in the innate immune response of the CNS to viral infection (96, 97). Different immune cells in the CNS respond to various viral stimuli. Microglia monitor the local environment and rapidly respond to widespread inflammatory stimuli, while astrocytes function as immune response cells and produce large amounts of inflammatory mediators (98). For example, during infection with Taylor mouse encephalomyelitis virus (TMEV), protein levels of ISG15 were elevated mainly in astrocytes and endothelial cells, whereas the protein levels of protein kinase R (PKR) were predominantly increased in microglia/macrophages, oligodendrocytes and neurons (49). Murine hepatitis virus (MHV) is a neurotropic coronavirus, and astrocytes and microglia produce type I IFNs (IFN-α and IFN-β), as well as interleukin (IL-6), TNF-α, IL-12, IL-1α, and IL-1β during experimental MHV infection in mice (99). HSV encephalitis (HSE) is a severe CNS infection caused primarily by HSV-1 and occasionally by HSV-2. Following infection with HSV-1, TLR2 and TLR4 are induced to activate simultaneously, and in turn, TLR2 forms a dimer with TLR1, or TLR6, which then induces IFN-β in neurons and IFN-α in astrocytes, and these IFNs subsequently induce the expression of ISGs such as viperin, CH25H (cholesterol-25-hydroxylase), oligoadenylate synthase2 (OAS2), latent RNase (RNase L), PKR, and IFIT1 (59, 60).

### Microglia

Microglia, a major source of type I IFNs, exert direct antiviral effects by producing type I IFNs to stimulate the expression of ISGs or act indirectly on other cells through type I IFNs to activate the corresponding signaling pathway (100). Microglia express various pattern recognition receptors (PRRs), including TLRs, RNA-sensing RLRs, and cytosolic DNA sensors that are important for virus defense. PRRs recognize viral-associated molecular patterns and induce type I IFN expression in microglia (27, 101). Cyclic GMP-AMP synthase (cGAS) binds to cytoplasmic dsDNA in microglia to produce cyclic GMP-AMP (cGAMP), which activates downstream stimulator of interferon genes (STING) and ultimately activates the transcription factor IRF3. In turn, IRF3 stimulates type I IFN production, and the resulting type I IFNs bind to the heterodimeric receptor IFNAR to initiate a signaling cascade, promote nuclear translocation of heterodimeric STAT1/2, and facilitate transcriptional activation of multiple ISGs (59, 60, 67, 90, 99). Depletion of CNS microglia *via* CSF1R inactivation has higher viral loads in mice infected with WNV (TX02 strain) or VSV, with increased mortality and viral tissue loads, indicating that microglia are critical for restricting virus transmission (102, 103). The phagocytic activity of microglia recruited *via* purinergic receptor P2Y12 signaling around infected neurons play an important role in CNS antiviral immunity. Analysis of temporal lobe specimens from patients with HSV-1 encephalitis reveals that there are approximately 1–3 activated microglia around each HSV-1-positive neuron and that P2Y12-positive microglia processes extend to HSV-1-positive cells (104). In addition, the number of microglia recruited to infected neurons was significantly reduced in a P2Y12-deficient mouse model (104). Studies have shown that microglia are the main producers of type I IFNs in viral infections of the CNS. Further studies found that in the mouse model of VSV encephalitis, the infected microglia were found to produce type I IFNs, which caused both infected and uninfected microglia to upregulate the expression of IRF7 and activate innate immunity, thus limiting the trans-synaptic transmission of VSV (105). Microglia also induce IFN and ISG expression by regulating the HSPA8/DNA-PK pathway independently of STING (106). Although phagocytosis of foreign pathogens by microglia is an essential component of neuroprotective immune defense to ensure the function of healthy neurons, excessive microglia activation leads to uncontrolled inflammation that exacerbates neuronal death, causing damage to brain tissue and cells (68, 107). In mice infected with JEV, microglia activation led to uncontrolled inflammation and neuronal death (108). JEV can promote viral replication by infecting microglia and upregulating miR-146a gene expression, inhibiting NF-κB activity, blocking the antiviral JAK/STAT signaling pathway, and downregulating antiviral ISGs (IFIT1 and IFIT2) (68). Phosphorylation of IFN regulators (IRF3 and IRF7) and STAT1/3 are inhibited in HIV-infected microglia, which suppresses the expression of several key anti-HIV ISGs (ISG15, IFIT1, GBP5, MX2, and viperin) (51). Taken together, these findings suggest microglia play an essential role in antiviral defense of the CNS and contribute to explaining how the virus invades microglia and results in a persistent infection.

### Astrocytes

Astrocytes have essential and significant functions in synaptic plasticity, regulation of the BBB, and maintenance of CNS homeostasis (109, 110). In viral infections, astrocytes play a key role in host defense by supporting a functional BBB, regulating glutamate homeostasis, and engaging in innate and adaptive immune response to viral infections (111). Recent studies have shown that microglia induce astrocytes proliferation through the expression of pro-inflammatory cytokines, including IL-1β, TNF, and IFN-γ (112, 113). During viral infection, astrocytes detect molecular changes in their extracellular environment and neighboring cells. Compared with microglia, astrocytes have low basal mRNA levels of PRRs and ISGs, and poorly induced *Ifn-β* mRNA following infection, but the upregulated various mRNAs in the IFN-α/β pathway of astrocytes to a higher extent than microglia, suggesting that the response of astrocytes to infection is delayed but stronger compared with that of microglia (114). Genetic astrocyte-specific deletion of the type I IFN receptor IFNAR in a mouse model of viral infection led to an increase of BBB permeability (115). Further studies revealed that abolition of astrocytic IFN-α/β signaling was followed by uncontrolled virus transmission and fatal encephalomyelitis, demonstrating the importance of the inducible IFN signaling pathway within astrocytes in limiting viral infection of the CNS (114). Imaizumi et al. reported that poly I:C upregulated the expression of IFIT2 and IFIT1 in astrocytes *via* the TLR3/IFN-β pathway and that the expression product IFIT1 positively regulated the expression of IFIT2, RIG-I, and MDA5 to enhance antiviral response (30). In addition, RNA interference (RNAi) knockdown of interferon-induced protein 35 (*IFI35*) resulted in a decrease in expression of poly I:C-induced *IFN-β*, *pStat1*, *Rig-1*, *Cxcl10*, and *Ccl5*, indicating that IFI35 may negatively regulate the astrocyte TLR3/IFN-β/pSTAT1/RIG-I/CXCL10/CCL5 axis and may partially regulate the innate immune response of astrocytes (116). Recent studies of CNS complications due to enterovirus 71 (EV71) infection have shown that in infected astrocytes, phosphorylated and non-phosphorylated STAT3 competes with STAT1 for binding to KPNA1, inhibits nuclear import of pSTAT1 and hinders the formation of the ISGF3 complex, leading to suppression of downstream ISG expression (117). Knockdown of STAT3 attenuated the suppressed IFN-mediated antiviral response to EV71 infection and led to a reduction in viral replication, demonstrating the role of STAT3 in maintaining the balance of inflammatory response in astrocytes and antiviral response in the CNS during infection (117). Borna disease virus (BDV) is a non-hemolytic RNA neurotropic virus, and replication of this virus is effectively blocked in transgenic mice expressing mouse IFN-α in astrocytes (118). Another study showed that rapid type I IFN response protected astrocytes from virus-induced cytopathic effects upon infection with flavivirus, tick-borne encephalitis virus (TBEV), JEV, WNV, and Zika virus (ZIKV), thus limiting the spread of these viruses (119). In addition, type I and type III IFN-independent antiviral pathways were found to be involved in the control of astrocytes during ZIKV infection (120). In summary, the intrinsic structural antiviral response of astrocytes combined with rapid induction of type I IFNs is instrumental in protecting astrocytes and inhibiting viral replication in the CNS (121).

### Neurons

Like microglia and astrocytes, neurons express multiple PRRs, produce innate immune cytokines such as type I IFNs following viral infection, and respond to cytokine stimulation to inhibit viral replication and increase cell survival (122–125). Previous studies have shown neuronal upregulation of key antiviral effector molecules and other ISGs in response to neurotropic virus infection, but the neurons produce a limited amount of IFN and express fewer ISGs compared with microglia (126). Delhaye et al. identified approximately 16% of IFN-producing cells corresponding to neurons, but only 3% of infected neurons produced IFN after infecting mice with two neurotropic viruses that primarily infect neurons (La Crosse virus and Theiler virus) (122). ISGs suppress viral replication by directly interrupting the viral life cycle or by stimulating the production of antiviral factors in infected and adjacent cells, but the effects vary considerably in different regions of the brain (127). Further studies have shown that the induction and response to ISGs vary considerably in diverse neuronal populations. In transgenic mice expressing IFN, the expression of typical IFN response marker MX1 was higher in CA1 and CA2 neurons in comparison with CA3 neurons in the hippocampal region (118). Lucas et al. discovered that the IFN-α-inducible protein 27 like 2A (*Ifi27l2a*), which is upregulated in the cerebellum, brainstem, and spinal cord after WNV infection, limits viral infection in these regions but not in other neurons and cells, implying that *Ifi27l2a* contributes to WNV innate immune restriction in certain cell types and tissue-specific manner (76). Furthermore, following LGTV infection in the CNS, the activity of viperin, an interferon-inducible protein that inhibits replication, effectively limits LGTV replication in the OB and brain but does not inhibit virus replication in the cerebellum (128). Viperin also reduced TBEV replication in primary cortical neurons and astrocytes *in vitro*, but not in cerebellar granule cell neurons (61).

Mutations in human TLR3 are essential in the development of human HSV-1 encephalitis (129–131). TLR3 deficiency impairs the cell-autonomous defense of IPSC-derived cortical neurons and oligodendrocytes against HSV-1 infection, but not that of trigeminal ganglion (TG) neurons, owing to TLR3 control of ISG mRNA expression levels induced in human pluripotent stem cell-derived cortical neurons but not TG neurons (132–134). It has also been reported that neurons at different developmental stages express different levels of ISGs in response to viral infection. During LACV-infected encephalitis, both LACV-infected neural precursor cells and mature neurons undergo apoptosis, but neuronal maturation increases the susceptibility of neurons to LACV-induced apoptosis because mature neurons express less ISGs compared to neural precursor cells (135). Unlike the CNS, the antiviral response of peripheral neurons, such as the dorsal root ganglion (DRG), is more dependent on the dual action of antiviral ISGs and autophagy activation. DRG produces only a small number of type I IFNs and does not effectively induce the production of ISGs (136).

### Oligodendrocytes

Oligodendrocytes have been shown to be less responsive to IFN in comparison with microglia (137). Mouse oligodendrocytes have lower basal expression levels of PRRs, IFN-α/β, ISGs, and kinases and transcription factors essential for IFN-α/β signaling and displayed a later expression of ISGs by comparison to microglia. Despite the fact that infection increases the expression of ISGs in both cell types, oligodendrocytes have a more limited expression profile and absolute mRNA levels compared with microglia. This limited antiviral response is associated with the inability to upregulate IκB kinase (IkappaB kinase or IKK) and IRF7 transcripts, both of which are required for amplification of the IFN-α/β response (138).

## Expression of ISGs in neurological diseases

### ISGs in neuroinflammation-related diseases

Upregulation of ISGs is associated with neuroinflammation-related diseases, including neuroinflammatory diseases such as multiple sclerosis (MS) (62) and neurodegenerative diseases such as AD (46), PD (139), and ALS (54). MS patients have elevated concentrations of the bile acid precursor 25-hydroxycholesterol (25-HC) in the cerebrospinal fluid (CSF), possibly as a result of the upregulation of the ISG CH25H in macrophages (62). 25-HC is mainly synthesized from cholesterol by CH25H, and has been shown to modulate inflammatory response and oxidative stress in normal or pathological nervous systems (140, 141). Wang et al. reported that ISG15 positively correlated with the degree of neuronal damage in an animal model of ALS with no obvious signs of inflammation, a model of cerebral ischemia, a model of brain injury induced by cortical shocks, and a mouse model of chronic neuronal damage induced by the viral protein HIV gp120, with high and significantly elevated ISG15 levels in areas of neuronal damage (54). This suggests that ISG15 may be a reliable biomarker of pathological changes in the CNS (54). Mutations in the ATM gene contribute to ataxia capillaries (A-T), a rare neurodegenerative and immunodeficiency disorder characterized by cerebellar ataxia capillaris, immunodeficiency, radiosensitivity, and cancer susceptibility. Studies have shown that in A-T, the level of ISG15 is significantly higher in the cerebellum than in the brain (55). In glucosylceramidase1 (GBA1) deficient mice (which causes Parkinson’s disease alpha-synuclein pathology), IFN-β levels are elevated in neurons, and ISGs are elevated in microglia (139). IFP35 is significantly upregulated in patients with untreated MS, demonstrating that IFP35 expression levels predict disease outcome and treatment response in MS (77). TLR3, which is primarily activated in innate immunity due to viral infection and induces the production of downstream ISGs (142), has also been found to be activated in alcohol-induced brain injury (143, 144). McDonough et al. reported TLR4-dependent upregulation of ISGs in ischemia/reperfusion-induced microglia (145). Meanwhile, in an AD model, activated microglia express ISGs, and the microglia are centered around amyloid-β (Aβ) plaques and accumulate in an age-dependent manner (46). Intracerebral injection of recombinant IFN-β activated microglia and eliminated complement C3-dependent synapses. Conversely, selective IFN receptor blockade effectively reduced ongoing microglia proliferation and synapse loss in AD models, demonstrating that ISGs are associated with a reduction in synapses (46). Aicardi-Gtières syndrome (AGS) is a severe inflammatory disease mimicking congenital infection with significant IFN production, characterized by chronic CSF lymphocytosis and elevated IFN-α levels, which can lead to severe neurodevelopmental disorders, spastic dystonia, and abnormal tetraplegia (146). ISGs, such as ISG15, viperin, and IFI27, are consistently elevated in patients with AGS, and these elevated ISGs are highly correlated with disease onset and progression (57). Mutations in adenosine deaminase (ADAR1) are crucial mechanisms for the development of AGS. In ADAR1-mutant mice, the expression of ISGs in neurons and microglia is selectively activated in a patchy manner, and the expression of *Isg15* in brain neurons with ADAR1 mutation is upregulated (147). It has also been reported that conditional deletion of ADAR1, specifically in mouse neural spinal cells, leads to overall peripheral nerve depigmentation and myelin loss, and that upregulation of ISGs precedes these defects, suggesting that ISGs may be involved in the production of such defects (148). Collectively, these studies have, in part, revealed a complex relationship between the IFN signaling pathway and neuroinflammation-related diseases.

### ISGs in neuropsychiatric disorders

In recent years, evidence has accumulated to show that ISGs play an important role in psychiatric symptoms caused by CNS disorders. Studies have shown an intrinsic link between type I IFN therapy and severe neuropsychiatric disorders, mainly major depression (149). Considerable evidence suggests that type I IFN is associated with psychiatric disorders, and that the production of type I IFNs as a result of TLR4 induced IRF3 activation and TLR7 induced IRF7 activation may be closely associated with IFN-mediated psychiatric disorders (93). Infant onset of RNaseT2-deficient leukoencephalopathy leads to cystic brain injury, multifocal white matter changes, brain atrophy, and severe psychomotor impairment. *Rnaset2^-/-^* mice exhibit upregulation of ISGs and IFNAR-I-dependent neuroinflammation (150). HIV-associated neurocognitive disorders (HAND) also show an upregulation of ISG15 (151). In addition, ISGs may be involved in neuronal and synaptic regulation. The upregulation of inflammatory cytokines induced by maternal immune activation (MIA) promotes ISG15 expression in the offspring’s brain, leading to neuronal dendritic lesions and depression-like behavior (56). In the hippocampus, ISG15 and Ubiquitin-specific peptidase 18 (USP18) mediate IFN-α-induced reduction in neurogenesis through upregulation of ISGylation-associated proteins UBA7, UBE2L6, and HERC5 (152). Adolescent mice injected with the viral mimic poly I:C had significantly increased expression of ISGs (IFIT2, PRKR, MX2, and IRF7) in the hippocampal dentate gyrus and exhibited behavioral deficits of impulse inhibition and impaired recognition of novel objects (58). In summary, upregulation of ISGs in the CNS may regulate various cellular functions and processes, such as neuronal survival and synaptic pruning, in a brain region-dependent manner (94).

## The roles of individual ISGs in CNS viral infections or neurological diseases

To date, type I IFNs remain the most potent, broad-spectrum antiviral agents. The treatment of IFN to cells induces a large set of ISGs that can prevent infection with many viral pathogens. There are currently more than 300 recognized ISGs, but the exact mechanisms of inhibiting virus replication have been identified only in a small subset of ISGs. Detailed mechanistic investigation of the functions of individual ISGs is complicated by the difficulty in dissecting particular processes in virus replication independently of one another. The responses of individual ISGs to different viral infections in different organs may also vary, and there are limited in-depth studies on the effects of individual ISGs on the nervous system. With continuous research in this field, the role of ISGs and their mechanisms are being elucidated. The following subsections summarize several representative ISGs found in viral infections and neurological diseases in recent years.

### ISG15 and ISGylation for the regulation of neurological diseases

As mentioned above, Wang et al. found that ISG15 positively correlated with the degree of neuronal injury in animal models of ALS with no obvious signs of inflammation, a model cerebral ischemia, a model of cortical shock-induced brain injury, and a mouse model of chronic neuronal injury caused by the viral protein HIV gp120, with low levels of ISG15 in unaffected areas and high levels of ISG15 in neuronal injury areas. In patients with ALS, elevated levels of ISG15 and ISGylation in the CSF were significantly higher in post-traumatic brain injury ALS compared with those in non-traumatic brain injury ALS (53). It is suggested that ISG15 may be a reliable biomarker of pathological changes in the CNS (54). In cells with mutations in ATM kinase, conjugated ISG15, but not the free form, antagonizes targeted degradation of the ubiquitin pathway, which may lead to progressive neurodegeneration in A-T patients (153). C-Type Lectin Domain Containing 16A (CLEC16A) has been shown to function in autophagy/mitochondrial autophagy and Clec16 knockdown leads to an inflammatory neurodegenerative phenotype similar to spinal cerebellar ataxia in mice. In the whole-body inducible knockout of Clec16a mice model, Clec16a expression was negatively correlated with IGS15 expression, and the expression of ISG15 in neuronal tissues was upregulated, suggesting that ISG15 may be a link between Clec16a and downstream autoimmune inflammatory processes (154). ISG15 binds to a number of key proteins and affects various pathophysiological processes in the CNS. After traumatic brain injury, ISG15 is rapidly elevated and binds covalently to myosin light chain kinase (MLCK), which may promote phosphorylation of the myosin light chain by MLCK and conversion of F-actin to stress actin, which is involved in BBB destruction by disrupting TJs, thus aggravating brain edema (155). IFN-β inhibits the MAPK signaling pathway and attenuates mechanical nociceptive hyperalgesia by elevating both free and conjugated ISG15, an effect that is increased in *ubp43^-/-^* mice lacking the key de-binding enzyme (156). Upregulation of MIA-induced inflammatory cytokines promotes ISG15 expression in the offspring brain, leading to neuronal dendritic lesions and depressive-like behavior through a mechanism of ISG15 inhibiting the ubiquitination of Rap2A by NEDD4 (an E3 ubiquitin ligase that ubiquitously inhibits Rap2A activity, leading to dendritic growth and depoliticization), thus inducing Rap2A accumulation (56). In contrast, upregulation of NEDD4 abolishes ISG15-induced dendritic damage (56). In a model of acute inflammation established by LPS-stimulated microglia, increased ISGylation maintained the stability of STAT1 and promoted a sustained immune response during inflammation (157).

### IFIT2 specifically limits neurological viral infections

Interferon-induced proteins with tetratricopeptide repeats (IFIT) are prominent ISGs, induced following type I IFN- or IRF3-dependent signaling, contribute to the antiviral defense of cells by binding directly to viral RNA or by binding to eukaryotic initiation factor 3 (eIF3) and preventing eIF3 from initiating the viral translational process (158). The human IFIT gene family generally consists of four members: IFIT1, IFIT2 (ISG54, p54), IFIT3 (ISG60, p60), and IFIT5 (ISG58, p58), whereas the mouse IFIT gene family encodes for three relevant genes: IFIT1, IFIT2 and IFIT3 (ISG49, p49), which are induced during IFN signaling pathway, viral infection or other PAMP recognition and have critical roles in host antiviral defense (159, 160). IFIT1 had an antiviral effect in human cytomegalovirus (HCMV)-infected human astrocytes isolated from the fetal brain, but not in HELFs (human embryonic lung fibroblast cells) (69). Recent studies have shown that IFIT2 primarily limits viral infection and protects mice from severe morbidity and mortality following infection with RABV (75), lethal VSV (67, 70), WNV (71), and Sendai virus (SeV) (72). IFIT2 acts as an antiviral in the CNS in several ways. Both VSV and EMCV infections cause neuroinvasive disease and induce IFN-β, IFIT1, and IFIT2 in the brain. However, IFIT2 only prevents VSV invasion of the brain and not EMCV invasion of the brain, suggesting that the antiviral response of IFIT2 in the CNS is virus-specific (67). In ***Ifit2^-/-^*** mice, effective VSV viral replication was restricted to the brain, and the absence of IFIT2 did not affect viral titers in other organs such as the liver or lungs, suggesting that IFIT2 can limit VSV invasion of the nervous system (67). In WNV-infected CNS, viral titers were higher in ***Ifit2^-/-^*** mice compared with those in WT mice only in the OB, cerebral cortex, brainstem, cerebellum, and spinal cord, and in cells with knockdown of IFIT2, increased WNV infection was observed only in cerebellar granule cells and dendritic cells, but not in macrophages, fibroblasts, or cortical neurons (71). Overall, these data suggest that IFIT2 has a crucial role in limiting viral infection in specific regions of the brain and in specific cell types. In experiments with RABV infection of the CNS, IFIT2 exerted antiviral effects predominantly at the level of viral replication and not as a mechanism to restrict viral entry/exit or transport of RABV particles *via* axons (75). Furthermore, IFIT2 can be involved in antiviral response by inducing and enhancing innate immunity. In neurotropic coronavirus MHV-RSA59 infection, IFIT2 promoted viral clearance by facilitating microglia activation and recruitment of NK1.1 and CD4 T cells to the brain (73). Further studies have shown that IFIT2 and IFIT3 function in a complementary and synergistic manner to restrict RABV in mouse-derived neuroblastoma cells (74). In MHV-induced encephalitis, IFIT2 is a positive regulator of IFNα/β expression rather than a direct antiviral mediator, with *Ifit2^-/-^* mice showing significantly reduced expression of IFN-α/β and the downstream ISG mRNAs (*Ifit1, Isg15, and Pkr*) (52).

### The role of IFITM3 in CNS infection and Alzheimer’s disease

The IFN-inducible transmembrane proteins (IFITMs) form a small family of IFN-inducible proteins and have two transmembrane structural domains. The IFITMs were shown to inhibit the cellular entry step of many enveloped viruses such as influenza A, dengue, Ebola, and SARS coronavirus (161). The human IFITM family consists of four proteins, IFITM1, IFITM2, IFITM3 and IFITM5, located on chromosome 11, among them IFITM1, IFITM2 and IFITM3 are well-known ISG proteins (162). IFITMs disrupt the entry of multiple enveloped viruses, and play a role in the transport of viral particles to lysosomes for degradation (163). SARS-CoV-2 infection was recently shown to increase IFITM3 protein expression (63), and in severe SARS-CoV-2 cases, IFITM3 levels are elevated in the frontal cortex and choroid plexus (65). IFITM3 may prevent pathogenesis by limiting early replication and transmission of α-virus in the brain and spinal cord (164). In patients with Rasmussen encephalitis (RE) caused by infection with HCMV viruses, IFITM3 was detected in the neurons of brain tissue, and there was colocalization of HCMV and IFITM3, suggesting that HCMV infection may induce IFITM3 expression in neurons and that IFITM3 can effectively inhibit HCMV infection and participate in the immune response to HCMV infection in RE brain tissue (64). Further studies found that the IFITM3 single nucleotide polymorphism (SNP) rs12252 correlated with the severity of disease caused by viral infection (165–167). The rs12252-C mutant protein IFITM3 ND21 was not flexible enough to effectively prevent the fusion of the virus with the endocytic membrane, which in turn reduced the ability of the immune system to defend against viral infection. Wang et al. found that subjects carrying IFITM3 rs12252 CC genotype were at increased risk of developing RE and were associated with rapid progression of RE disease (64). In addition, the rs12252-C allele was recently reported to be associated with disease severity in patients with SARS-CoV-2 (168). In conclusion, the IFITM3 rs12252-C allele is strongly associated with the severity of some viral infectious diseases (169).

IFITM3 mRNA expression in the cortex and hippocampus is significantly positively correlated with age (ranging from 20 years to 70 years) in humans, according to genotype-tissue expression cohorts (66). IFITM3 protein levels, Aβ production (Aβ42 and Aβ40), and the amount of active IFITM3-γ-secretase were increased in the aging WT mouse brains (66). IFITM3 expression is upregulated in astrocytes and microglia in the brains of the 5xFAD Alzheimer’s disease mouse model, and IFITM3 mRNA and IFITM3 protein are expressed in neurons (66). Pro-inflammatory cytokines (IFN-α or IFN-γ, IL-6, and IL-1β) increase Aβ production in neurons and astrocytes by increasing the formation of IFITM3 protein and active IFITM3-γ-secretase complexes (66). Recently, SARS-CoV-2 has been reported to increase IFITM3 protein (63), and IFITM3 levels are elevated in the frontal cortex and choroid plexus in severe SARS-CoV-2 cases (65). Hur et al. concluded that different inflammatory conditions, such as viral infection and aging, can induce the release of pro-inflammatory cytokines from astrocytes and microglia, which in turn elevate the expression of IFITM3 in neurons and astrocytes, and IFITM3 binds to active γ-secretase complexes, increasing Aβ production and increasing the risk of AD (66).

## Conclusion

This review highlights the ISGs involved in resisting the neurotropic viral invasion of the CNS and the mode of activation of these ISGs in viral-infected CNS cells. Furthermore, the expression characteristics of ISGs in the development of CNS disorders are discussed. At last, we summarize in detail several mechanisms of action of individual ISGs in the CNS that have been more studied in recent years. The IFN signal pathways induce hundreds of ISGs to exert antiviral and other physiopathological effects. ISGs are a large family, and many more are still waiting to be identified. Most studies have focused on ISG as a marker of activation of the innate immune response to IFN, whereas the mechanisms of ISG in the pathophysiological response of the CNS remain unclear and need to be investigated in depth. Thus this review summarizes the current research on ISGs in CNS and indicates possible directions for future research. With the development of technologies such as CRISPR-Cas9 gene editing as well as genome-wide RNA-seq and deep proteomics (170), research on the antiviral effects of individual ISG, as well as its other functions in CNS diseases, is expected to evolve rapidly. An improved understanding of the functions of individual ISGs will facilitate the development of ISG-based therapies. Consequently, ISGs may exhibit promise as potential clinical biomarkers as well as therapeutic targets.

## Author contributions

RL, YY, CB, and WD conceived the perspective of the work. RL, HL, YY, CB, and WD drafted the manuscript. HL, XL, and CL designed the figure. YZ, SG and JX assisted in collecting and organizing the literature. All authors revised and approved the final version of the manuscript. All authors contributed to the article and approved the submitted version.

## Funding

This work was supported by the National Natural Science Foundation of China (31871031, 32170968, WD), the Fund of Key Laboratory of Medical Electrophysiology in 2021 (KeyME-2021-01, YY), and China Postdoctoral Science Foundation (2021M692700, YY).

## Conflict of interest

The authors declare that the research was conducted in the absence of any commercial or financial relationships that could be construed as a potential conflict of interest.

## Publisher’s note

All claims expressed in this article are solely those of the authors and do not necessarily represent those of their affiliated organizations, or those of the publisher, the editors and the reviewers. Any product that may be evaluated in this article, or claim that may be made by its manufacturer, is not guaranteed or endorsed by the publisher.

## Glossary

**Table:**

IFN	Interferon
ISGs	Interferon-stimulated genes
CNS	Central nervous system
BBB	Blood-brain barrier
JAK	Janus tyrosine kinase
STAT	Signal transducer and activator of transcription
ISGF3	IFN-stimulated gene factor 3
TYK2	Tyrosine Kinase 2
IRF9	Interferon regulatory factor 9 (IRF9)
ISREs	Interferon stimulatory response elements
GAF	γ-activated factors
GAS	γ-activated sequences
IRFs	Interferon regulatory factors
NF-kB	Nuclear factor k B
RIG-I	Retinoic acid-inducible gene I
MDA5	Melanoma differentiation-associated gene 5
RLRs	RIG-I-like receptors
IFIT1	Interferon-induced protein with tetrapeptide repeats 1
WT	Wild-type
SINV	Sindbis virus
HSE	Herpes simplex encephalitis
TRIF	Toll-interleukin-1 receptor domain-containing adaptor-inducing interferon-β
TBK-1	TANK-binding kinase 1
TLR3	Toll-like receptor 3
TRAF3	Tumor necrosis factor receptor-associated factor 3
AD	Alzheimer's disease
PD	Parkinson's disease
ALS	Amyotrophic lateral sclerosis
BCSFB	Blood-cerebrospinal fluid barrier
HSV-1	Herpes simplex virus type 1
VZV	Varicella-zoster virus
JEV	Japanese encephalitis virus
RABV	Rabies virus
BMECs	Brain microvascular endothelial cells
TJ	Tight junction
LPS	Lipopolysaccharide
HIV	Human immunodeficiency virus
NMJs	Neuromuscular junctions
OB	Olfactory bulb
LGTV	Langat virus
WNV	West Nile virus
VSV	Vesicular Stomatitis Virus
CMV	Cytomegalovirus
IFNAR	IFN-α/βreceptor
CAMs	CNS-associated macrophages
TMEV	Taylor mouse encephalomyelitis virus
PKR	Protein kinase R
MHV	Murine hepatitis virus
HSE	HSV encephalitis
CH25H	Cholesterol-25-hydroxylase
OAS	Oligoadenylate synthase
RNase L	Latent RNase
PRRs	Pattern recognition receptors
cGAS	Cyclic GMP-AMP synthase
cGAMP	Cyclic GMP-AMP
STING	Stimulator of interferon genes
RNAi	RNA interference
IFI35	Interferon-induced protein 35
EV71	Enterovirus 71
BDV	Borna disease virus
TBEV	Tick-borne encephalitis virus
ZIKV	Zika virus
Ifi27l2a	Interferon alpha-inducible protein 27 like 2A
DRG	Dorsal root ganglion
IKK	IkB kinases
MS	Multiple sclerosis
25-HC	25-hydroxycholesterol
CSF	Cerebrospinal fluid
A-T	Ataxia capillaries
GBA1	Glucosylceramidase1
AGS	Aicardi-Gtières syndrome
ADAR1	Adenosine deaminase acting on RNA1
HAND	HIV-associated neurocognitive disorders
MIA	Maternal immune activation
CLEC16A	C-Type Lectin Domain Containing 16A
MLCK	Covalently to myosin light chain kinase
HCMV	Human cytomegalovirus
HELFs	Human embryonic lung fibroblast cells
SeV	Sendai virus
IFITMs	IFN-inducible transmembrane proteins
RE	Rasmussen encephalitis
SNP	Single nucleotide polymorphism
SAMHD1	Sterile alpha motif and histidine/aspartic domain-containing protein 1
MX2	Myxovirus resistance protein 2
VSV	Vesicular stomatitis virus
KPNA1	Karyopherin-alpha1
CXCL10	C-X-C motif chemokine ligand 10
CCL5	Chemokine (C-C motif) ligand 5
IPSC	Induced pluripotent stem cells
IFP35	IFN-induced protein 35
MAPK	Mitogen-activated protein kinase
